# The bear circadian clock doesn’t ‘sleep’ during winter dormancy

**DOI:** 10.1186/s12983-016-0173-x

**Published:** 2016-09-17

**Authors:** Heiko T. Jansen, Tanya Leise, Gordon Stenhouse, Karine Pigeon, Wayne Kasworm, Justin Teisberg, Thomas Radandt, Robert Dallmann, Steven Brown, Charles T. Robbins

**Affiliations:** 1 Department of Integrative Physiology and Neuroscience, College of Veterinary Medicine, Washington State University, Mailstop 7620, Veterinary and Biomedical Research Bldg., Room 205, Pullman, WA 99164-7620 USA; 2 Department of Mathematics and Statistics, Amherst College, Amherst, MA 01002 USA; 3 Foothills Research Institute, Hinton, AB T7V 1X6 Canada; 4 U.S. Fish and Wildlife Service, Libby, MT 59923 USA; 5 Institute for Pharmacology and Toxicology, University of Zürich, Zürich, 8057 Switzerland; 6 School of the Environment, Washington State University, Pullman, WA 99164 USA; 7 Present address: Warwick Medical School and Warwick Systems Biology Centre, University of Warwick, Gibbet Hill Road, Coventry, CV4 7AL UK

## Abstract

**Background:**

Most biological functions are synchronized to the environmental light:dark cycle via a circadian timekeeping system. Bears exhibit shallow torpor combined with metabolic suppression during winter dormancy. We sought to confirm that free-running circadian rhythms of body temperature (Tb) and activity were expressed in torpid grizzly (brown) bears and that they were functionally responsive to environmental light. We also measured activity and ambient light exposures in denning wild bears to determine if rhythms were evident and what the photic conditions of their natural dens were. Lastly, we used cultured skin fibroblasts obtained from captive torpid bears to assess molecular clock operation in peripheral tissues. Circadian parameters were estimated using robust wavelet transforms and maximum entropy spectral analyses.

**Results:**

Captive grizzly bears housed in constant darkness during winter dormancy expressed circadian rhythms of activity and Tb. The rhythm period of juvenile bears was significantly shorter than that of adult bears. However, the period of activity rhythms in adult captive bears was virtually identical to that of adult wild denning bears as was the strength of the activity rhythms. Similar to what has been found in other mammals, a single light exposure during the bear’s active period delayed subsequent activity onsets whereas these were advanced when light was applied during the bear’s inactive period. Lastly, in vitro studies confirmed the expression of molecular circadian rhythms with a period comparable to the bear’s own behavioral rhythms.

**Conclusions:**

Based on these findings we conclude that the circadian system is functional in torpid bears and their peripheral tissues even when housed in constant darkness, is responsive to phase-shifting effects of light, and therefore, is a normal facet of torpid bear physiology.

**Electronic supplementary material:**

The online version of this article (doi:10.1186/s12983-016-0173-x) contains supplementary material, which is available to authorized users.

## Background

In the face of predictable periods of food scarcity animals have adopted a variety of survival strategies [1–3]. Two of these: hibernation and torpor, conserve energy through metabolic suppression and lowering of Tb to varying extents [4–7]. Biological rhythms (e.g. circadian, circannual) of Tb and body mass are important to hibernation [8–17]. The temperature dependence of torpor/arousal cycles has also been confirmed in several species [18]. Despite these observations, a substantial body of evidence indicates that circadian rhythmicity is lost during hibernation. For example, studies in European hamsters (*Cricetus cricetus*) demonstrated that the molecular clock “stops ticking” during hibernation [19]. Denning American black bears (*Ursus americanus*), which express only shallow torpor (3–5 °C lower Tb [3, 20]), may also have suppressed the ability to express circadian rhythms, [5, 20] and instead replaced these with multi-day cycles [21].

Because the brain contains the master circadian pacemaker in the suprachiasmatic nucleus (SCN), its role in hibernation has received considerable attention [15, 17, 22]. Those studies revealed that ablation of the SCN did not prevent the expression of activity and Tb rhythms during deep torpor in all animals, despite the animals being arrhythmic during euthermia. Other work also demonstrated that these rhythms, along with neural action potentials, are absent at Tbs between 0 and 16 °C [23, 24]. Yet, data indicating that the SCN of hibernators continues to be metabolically and physiologically active at even relatively low temperatures [22, 25–27], suggests the possibility that the SCN is capable of providing functional oversight or, alternatively, has other non-circadian roles during hibernation [10].

For circadian rhythms to be adequately characterized requires that subjects be held in constant environmental conditions of light and temperature thereby eliminating the major entrainment cues (*Zeitgeber*), especially light [28, 29]. Light resets the clock each day and keeps the animal’s physiology synchronized to local time thus matching that of the earth’s rotation (i.e., 24 h) [30, 31]. Constant environmental conditions have been used in some, but not all, hibernation/torpor studies (reviewed in [10]).

We recently reported that captive grizzly bears (*Ursus arctos horribilis*) housed in constant light (LL) and ambient temperature (Ta) conditions expressed free-running circadian rhythms of activity during winter dormancy [32]. That the period of these rhythms reverted to precisely 24 h (i.e., matched the natural environmental light:dark cycle period) when bears were exposed to Ta and lighting conditions or to fixed photoperiods was interpreted as evidence that the circadian clock was responsive to light cues even during this time [32]. Yet, how light and the circadian system interact during the long winter dormancy experienced by denning bears has not been fully elucidated. Moreover, the benefit (if any) of maintaining rhythmicity and possibly light entrainment during winter dormancy remains to be determined.

It has become abundantly clear that loss of synchronization (entrainment) to environmental light:dark cycles such as occurs during jet-lag or with shift work results in increased incidence of metabolic disturbances [33–36]; this suggests that rhythmicity and entrainment are both beneficial to overall energy homeostasis [37]. Indeed, energy balance is maintained within rather narrow limits due the coordinated actions of central and peripheral clocks (see [34] for Review) leading to the current hypothesis that rhythmicity facilitates metabolic efficiency [38]. Further support for this comes from studies demonstrating that disruption of peripheral clocks in the liver and pancreas are sufficient to disrupt whole-body glucose homeostasis [39, 40]. Thus, peripheral clocks serve as an independent yet important node in the metabolic machinery; but whether this node is functioning in all hypometabolic states is less clear.

Given the variable outcomes of studies examining the circadian system in hypometabolic states we sought to clarify this issue in torpid bears. To this end, we extended our previous work to explore several other features of the circadian system, including: 1) its free-running period in constant darkness (DD) in an effort to more closely mimic photic conditions presumably experienced in a natural bear den, 2), the ability of light to phase-shift rhythms - a basic property of the circadian clock, and 3) the integrity of the molecular clock in peripheral tissues. We also used data collected from wild bears to corroborate certain aspects of our captive bear studies.

## Results

### Confirmation of winter dormancy

**Fig. 1:**
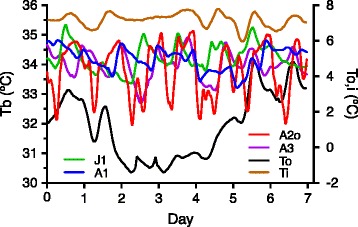
Representative Tb profiles of 4 bears (3 adults A1, A2o, A3) and 1 juvenile (J1) during the week of Jan 24, 2013. Also shown for comparison are: 1) the local environmental (outdoor, To) temperatures recorded at the Pullman regional airport located 1.5 miles (2.4 km) from the WSU Bear Center (PUW; data downloaded from http://www.ncdc.noaa.gov), and 2) the indoor isolation building temperatures (Ti) for the same period. Captive bears A1, A3, J1 were housed indoors under constant conditions while bear A2o was housed under naturally fluctuating light and temperature conditions

**Fig. 2:**
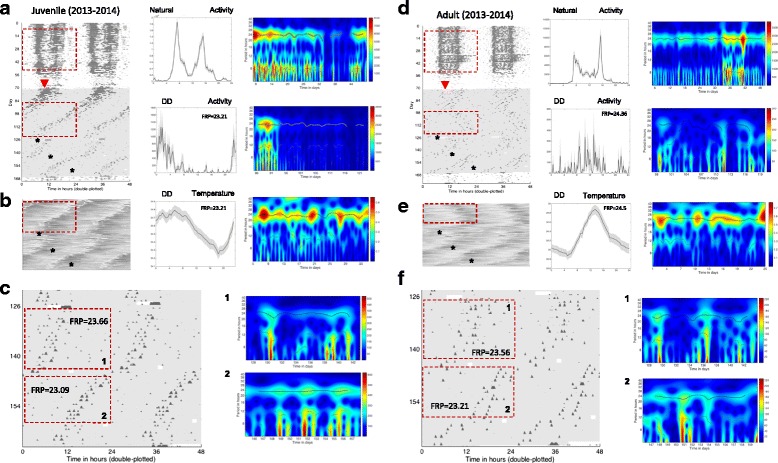
**a**,**d** Actogram- and **b**,**e** Tempogram-style plots from a captive juvenile (*male*) and adult (*female*) grizzly bear. Each black mark represents and activity bout or temperature change (see Methods for details). Actograms and tempograms are double-plotted to more easily observe trends across days. Thus, each row represents two days of data with the second day re-plotted on the row underneath the previous day and so on. Regions indicated by the red dashed boxes are re-plotted in the middle column as daily mean activity or Tb profiles (averaged over the time-frame corresponding to the boxed region) and in the right column as scalograms. The scalograms reflect the day-to-day changes in period length (*black/white* horizontal line near 24 h period) and rhythm amplitude (heat map color). Actogram data extend from the pre-dormancy period (white background) through winter dormancy (*gray background*) whereas the tempogram data begin at dormancy (*red triangle*) onset and extend for various times in winter dormancy. **c**,**f** Expanded view of the winter dormancy periods shown in Panels **a** and **d** illustrating the times when light pulses where applied (*white boxes*). Boxed areas are re-plotted as scalograms in the right column and correspond to each numbered box. Asterisks indicate the onset of light pulses. FRP – Free running period

Bears were confirmed to have entered winter dormancy based on low Tb (Fig. 1) and dramatic reduction in activity levels (>90%; Fig 2a, d; see also [32, 41]; Table 1 and 2). To further confirm the torpid state of our bears we measured Tb in 6 of the 9 bears used in the current study (4 adults, 2 juveniles) during the active (May-October) and dormant periods (January). A significant reduction in average (±SD) Tb from 37.0 °C (0.5) to 34.4 °C (0.9), *p* < 0.001 (Welch’s *t* test) was observed. The temperatures obtained during hibernation between the two groups were virtually identical. The relationship between Tb and Ta (outdoor and indoor) over a 7-day period in late January, 2013 is shown in Fig. 1. In contrast to the large fluctuations in outdoor temperature, indoor temperatures remained tightly regulated around the 7 °C set point. Tb of all the indoor bears fluctuated in a daily manner that was distinct from the daily Ta fluctuations while that of the single bear housed under natural conditions appeared to cycle somewhat with outdoor temperatures (Fig. 1). Additionally, while the maximum Tb in bears housed indoors was virtually identical to that of the single bear (A2o, Fig. 1) housed under natural daylight and temperature conditions, the minimum temperature in this bear was always 1–1.5 °C lower as was his average mean Tb during winter dormancy (Table 2). Body weights of all bears immediately prior to entering winter dormancy are listed in Additional file 1: Table S1.

**Table 1:** Characteristics of activity rhythms in bears during winter dormancy

	CAPTIVE				
	DD All [12]	DD Juvenile [5]	DD Adult [7]	Ambient [6]	Wild [10]
Average Activity (Counts/min)	196 ± 5	293 ± 73^a^	90 ± 20^b^	34 ± 19^b^	74 ± 3^b^
Period (h)	23.97 ± 0.11	23.75 ± 0.10^a,†^	24.19 ± 0.08^b,†^	23.99 ± 0.07^c^	24.03 ± 0.02^b,c^
Acrophase (h)	15.74 ± 1.39	14.58 ± 2.02	17.29 ± 1.81	12.53 ± 0.74	13.71 ± 0.41
Strength (%)	9 ± 1	10 ± 2^a^	7 ± 1^a,c^	17 ± 3 ^a,b^	6 ± 1^a,c^
Variability (h)	1.07 ± 0.1	1.06 ± 0.13^a^	1.09 ± 0.16^a,b^	1.2 ± 0.09^a,b,c^	1.79 ± 0.12^d^
Amplitude (Counts/min)	272 ± 104	425 ± 13^a^	94 ± 13^b^	45 ± 9 ^b^	78 ± 5^b^

^*a,b,c,d*^Different letters within a row are significantly different from each other, *p* ≤ 0.05

^†^Significantly different from 24 h (one sample *t*-test, *p* < 0.05)

Numbers of animals are indicated in brackets

Values are means ± SEM

**Table 2:** Characteristics of body temperature (Tb) rhythms in captive bears during winter dormancy

	CAPTIVE			
	DD All [9]	DD Juvenile [5]	DD Adult [4]	Ambient [1]
Average Tb (°C)	34.4 ± 0.1	34.4 ± 0.1	34.3 ± 0.1	33.7
Min (°C)	31.7 ± 0.5	31.9 ± 0.9	31.5 ± 0.3	30.1
Max (°C)	36.3 ± 0.2	36.1 ± 0.3	36.5 ± 0.4	35.7
Period (h)	23.9 ± 0.09	23.8 ± 0.07^a^	24 ± 0.07	24
Acrophase (h)	17.8 ± 0.96	19.4 ± 1.8	15.3 ± 1.47	12.6
Strength (%)	66 ± 6	64 ± 10	68 ± 5	53
Variability (h)	1.3 ± 0.07	1.2 ± 0.08	1.5 ± 0.02	1.4
Amplitude (°C)	0.37 ± 0.03	0.4 ± 0 .03	0.4 ± .05	0.4

^a^Significantly different from 24 h (one sample *t*-test)

Numbers of animals are indicated in brackets

Values are means ± SEM

### Circadian rhythms

**Fig. 3:**
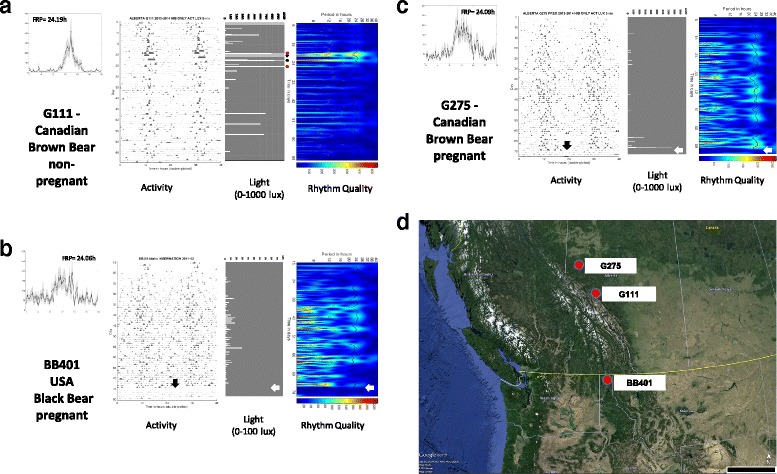
Daily (24 h) activity profiles and actograms (*left two columns*) and light intensity plots and scalograms (*right two columns*, both plots rotated 90° CW) illustrating the temporal relationship among the three parameters. Data from three female bears (**a**,**b**,**c**) with their corresponding den locations shown in Panel **d**

Circadian rhythms of activity and Tb were detected in all groups of bears based on wavelet transforms and maximum entropy spectral analysis (MESA) (Figs. 2 and 3; Table 1). Specific features of these rhythms differed depending on group (age, wild vs. captive), but no feature was deemed arrhythmic in any analysis, with one possible exception (see Discussion, Additional file 2: Figure S4). We observed a significant main effect of group on rhythm period (i.e., length) (F (3,34) = 12.31, *p* < 0.0001). Activity rhythms began their free-run in constant darkness (DD) in phase with their rhythms under natural conditions (Figs. 2 and 3a,d – left panels). This was characterized by activity bouts beginning slightly earlier each day (for a period <24 h) or later each day (for a period >24) (Figs. 2 and 5a,b; Table 1). Because temperature loggers were only implanted when the bears entered winter dormancy it was not possible to assess the phase at which their free-run began. Nevertheless, the close phase relationship between Tb and activity rhythms suggests a similar onset. Analysis of activity data revealed that the free-running period (FRP) of captive bears in DD did not differ from wild denning bears (Table 1). The average period of circadian activity rhythms in captive torpid bears housed in DD also did not differ significantly from that of captive bears exposed to Ta and photoperiod (Table 1). Interestingly, the period of juvenile activity rhythms (≤4 years. old) was significantly shorter than that of adults in DD (Table 1, Holm-Sidak post-hoc test, *p* < 0.0001). The FRP of juvenile bears was also significantly shorter than 24 h (1 sample *t*-test, t (9) = 3.632, *p* = 0.0055) whereas that of the of adults was significantly longer than 24 h (1 sample *t*-test, t (6) = 2.530, *p* = 0.0447). The period of Tb rhythms in DD was similar to that of activity rhythms (mean = 23.91 ± 0.11 h; juvenile = 23.81 ± 0.22 h, adult = 24.0 ± 0.14 h, Fig. 2). However, unlike activity rhythms, the period of the Tb rhythms did not differ between juvenile and adult bears (t (7) = 1.515, *p* = 0.16). The period of Tb rhythms also did not differ significantly from 24 h. Tb was not measured in wild bears.

The overall timing of daily activity and Tb rhythm maxima (acrophase) was similar (circadian time (CT) 14.9 ± 1.6 h vs. 13.8 ± 1.1 h, respectively; t (23) = 0.535, *p* = 0.598). However, the peaks occurred later in DD than under ambient conditions for adult bears (17.3 ± 1.8 h for DD adults vs. 11 h for a single adult bear exposed to ambient conditions receiving a temperature logger; 1 sample *t*-test, t (5) =3.470, p = 0.0178). The acrophases of Tb and activity rhythms were similar under ambient conditions (12.8 ± 1.2 h vs. 12.6 h; N = 1 for ambient Tb).

**Fig. 4:**
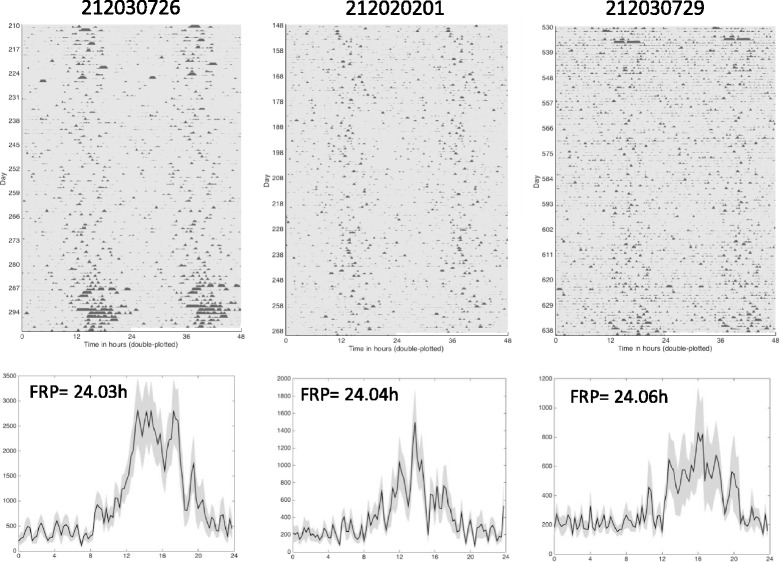
Actograms and corresponding daily profiles of three denning grizzly bears (one subadult male, one subadult female and one adult male) in the U.S.

Rhythm strength varied among bear groups and with parameter measured. Thus, for all torpid bears isolated from direct human disturbance (DD and Wild denning) significantly weaker rhythms (by 50–65 %, DWT calculation vs. active season (Fig. 2) were expressed compared to captive bears housed under ambient conditions and exposed to normal human disturbance occurring at the WSU Bear Center (main effect of group (F (3,33) = 6.738, *p* = 0.0011; Table 1). In addition, Tb rhythms were always significantly stronger than activity rhythms (66 % vs. 9 %, respectively, (t (9) = 9.895, *p* < 0.0001) but these did not differ between age groups (t (8) = 0.3148, *p* = 0.761; Table 2). Analytical waveform transform (AWT) analysis allowed us to examine the instantaneous period of activity and Tb rhythms to estimate their stability over time. The results revealed that circadian rhythm stability decreased significantly ((F 3,34) = 8.781, *p* = 0.0002) for all torpid bears when compared to the 30 days preceding onset of torpor (variability = 0.55 ± 0.051 h, *p* ≤ 0.01 vs. captive bears). The period of circadian rhythms occasionally changed from slightly less than 24 h to slightly greater than 24 h spontaneously in DD within a season - an extreme example is shown in Fig. 2d from an adult female bear (the change in rhythm period occurs around day 98). Minor changes appearing as ‘scalloping’ similar to those previously reported for grizzly bears [32] were more typically observed. Actograms of wild Candadian and U.S. grizzly bears also revealed slight scalloping in some, but not all, cases (Figs. 3 and 4).

The amplitude of activity rhythms varied significantly among groups (F(3,20) = 4.867, *p* = 0.0106). Juveniles had greater amplitudes than adults (Holm-Sidak post-hoc test, *p* = 0.0479) and bears exposed to ambient conditions (Holm-Sidak post-hoc test, *p* = 0.0208; Table 1). However, the amplitude of activity cycles of torpid adult bears in DD was similar to that of wild denning bears (Table 1). Amplitudes of Tb rhythms were only determined in captive bears and these did not differ between juvenile and adult bears (t (9) = 0.02402, *p* = 0.4464)). Activity rhythms and Tb rhythms always maintained a close temporal relationship (average coherence = 0.93 ± 0.01). This relationship did not differ between age classes (both = 0.93; Fig. 2). As expected, Tb rhythms lagged behind activity yielding a phase difference of -5.81 ± 0.84 h; this lag time also did not differ among age classes (juvenile = -6.42 ± 0.89 h vs. adults = -4.84 ± 0.67 h, t (9) = 1.365, *p* = 0.2055).

Mean (±SD) Ta in the isolation building were similar between different years of study (winter of 2013–2014: 7.2 ± 1.1 °C; winter of 2014–2015: 7.7 ± 1 °C) based on iButton measurements collected at 30 min intervals. In the first winter of the studies (2012–2013) room temperature averaged 7.5 ± 1 °C based on weekly temperature measurements made using a calibrated digital thermometer. The average daily variation in room temperature was 0.8 °C in 2013–2014 and 0.9 °C in 2014–2015 seasons.

### Light responses

**Fig. 5:**
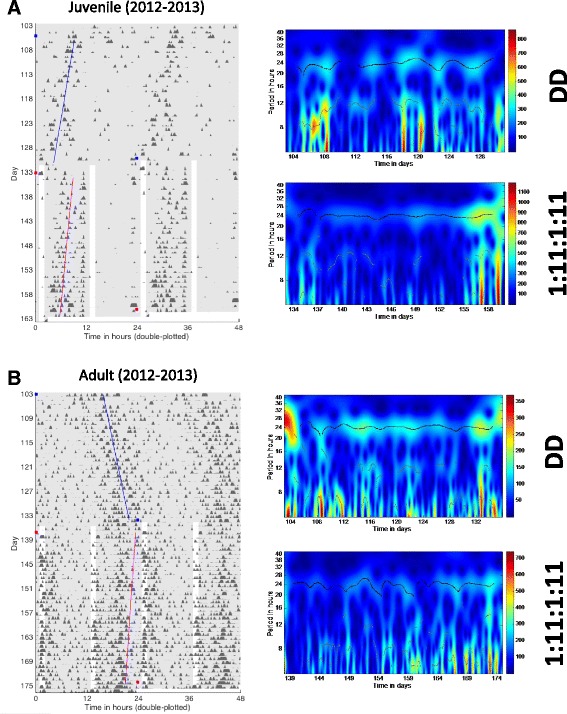
Actograms and scalograms of a juvenile (**a**) and adult (**b**) captive grizzly bear during winter dormancy under DD conditions or when exposed to a symmetrical (1 h light/11 h dark) twice daily light regimen (1:11:1:11). *Blue* and *magenta* lines represent the fitted midpoints of activity during each successive period. These are not the same bears shown in Figs. 1 and 2. Note the shorter FRP in the juvenile bear (*blue line angled left*) compared to the longer FRP in the adult bear (*blue line angled right*); a 24 h FRP would be represented by a completely vertical line

We exposed torpid bears to light in several ways to probe the sensitivity of the circadian system to this potent entrainment (synchronizing) agent. Light applied symmetrically as two one-hour pulses each 12 h apart (i.e., 1:11:1:11 photoperiod) resulted in the consolidation of activity after free-runs for both juvenile and adult bears (Fig. 5). The period of activity rhythms (23.91 h) under these photic conditions did not differ significantly from the 24 h experimental photic treatment (1 sample *t*-test *p* > 0.05); however, it did differ significantly from a 12 h cycle period (1 sample *t*-test *p* < 0.01), indicating entrainment had occurred, but to only one of the cycle frequencies. Specifically, adult bears exhibited a shortening of their prior free-running period (from 24.19 h to 23.87 h; t (4) = 4.950, *p* = 0.0078, *N* = 3)) whereas in juvenile bears (*N* = 2) the period lengthened from 23.75 h to. 23.95 h.

**Fig. 6:**
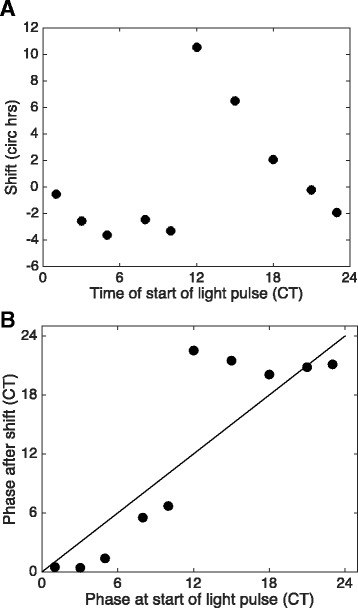
Phase-Response (**a**) and Phase-Transition (**b**) plots for all captive bears exposed to light pulses (1–4 h) in two consecutive years. The phase-response plot illustrates the relationship between the timing of the light pulse and shift in activity observed for several days after the light pulse was applied. The phase-transition plot illustrates the relationship between the ‘old’ phase (when light was applied) versus the ‘new’ phase (following the light exposure)

In contrast to the ‘skeleton’ photoperiod used above, application of only a single daily light pulse caused activity and temperature rhythms to shift in different ways depending on the time of circadian day that light was applied. The results are summarized as a phase-response curve in Fig. 6a. Thus, activity onsets were *delayed* when light was applied during the bear’s active period (circadian time (CT) 0-12) whereas activity onsets were *advanced* when light was applied during the inactive period (CT 12-24). Another way to depict the effect of light pulses on activity is shown in Fig. 6b where activity onset during the ‘old’ phase (determined on the day of the light pulse) is compared to activity onset during the ‘new’ phase (after the light). This so-called ‘phase transition’ plot revealed that when light is applied at virtually any time during the bear’s inactive period (CT12-24) the clock was always strongly reset to approximately the same time (CT22) corresponding to early morning (Fig. 6b). In contrast, light exposure during the active period produced a relatively proportional delay when compared to the original phase (Fig. 6b). The differences were not associated with longer light pulses because large advances were observed with 1 h light exposure (see Fig. 2c,f). Rhythm period was not always stable following a light exposure. This was observed in both captive bears in DD and in wild bears (Figs. 2 and 3); however, in the case of wild bears this was only visible on scalograms as small deviations (Fig. 3a-c); these events were not further analyzed due to methodological limitations.

We discovered that natural bear dens were periodically exposed to light during winter dormancy (Fig. 3a-c light plots) based on light sensors applied to collars on three wild bears (2 grizzly, 1 black) located in different ecosystems with different latitudinal gradients (Fig. 3d). The average (±SD) daily light exposures during the first 70 days of hibernation for which reliable activity and light data could be obtained due to battery life limitations were: Bear G111 = 4.32 (63.98) lux; Bear G275 = 0.67 (6.21) lux, Bear BB401 = 0.23 (1.08) lux). Light intensity (Additional file 3: Figure S1A) and duration (Additional file 4: Figure S2) were also found to vary dramatically among dens (range 5–85 min in Canadian brown bears and 15–800 min in the Idaho black bear). Light exposures were often interspersed by days to weeks of very low light (<5lux). We observed a noticeable effect of light in the natural den on the amplitude of activity rhythms when examining scalograms (Fig. 3a-c). Here, light exposure nearly always resulted in a dramatic increase in rhythm amplitude (e.g., Bear G111, Fig. 3a). The highest activity counts were also often associated with the most intense light exposure, although increased activity sometimes was observed without intense light, especially in the two bears that gave birth in the den (G275, BB401) (Additional file 3: Figure S1A). Light exposure seemed to occur most frequently around noon and was often associated with the largest activity bouts (Additional file 3: Figure S1B). Interestingly, activity rhythms disappeared entirely after parturition for the two females who gave birth in their dens at night (Fig. 3b,c arrows); rhythms then either remained absent (BB401) or disappeared after brief reappearance (G275) (Additional file 5: Figure S3A,B), respectively.

### In vitro rhythms

**Fig. 7:**
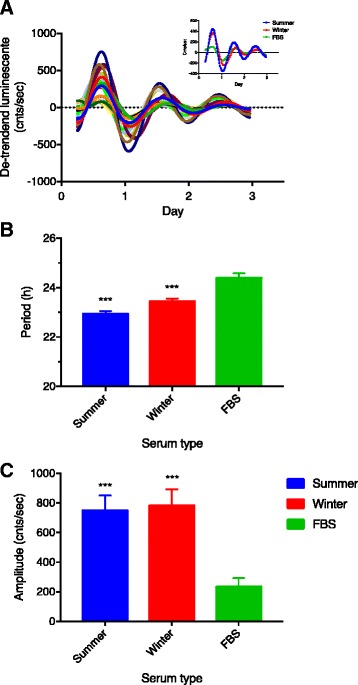
**a** Luminescence tracings of individual bear fibroblast cultured under different serum conditions. Fibroblasts were infected with a lentivirus construct containing the mouse Bmal1 promoter linked to the luciferase gene (see Methods for more details). Cultures are rhythmic but peaks occur at different times depending on the serum condition used. Inset represents the mean traces under each serum condition. **b**, **c** Summary data for in vitro rhythm period (B) and amplitude (C) of traces shown in A. ***P < 0.0001 vs FBS

Luminescence rhythms were expressed in fibroblasts obtained from four dormant captive juvenile bears and cultured at 37 °C (Fig. 7a). The rhythm period was longer than 24 h when cells were cultured under standard (i.e., 10 % FBS) conditions (Fig. 7b; Table 3). However, when the cells were cultured in the presence each bear’s own serum (10 %; obtained during either the active season or winter dormancy) the circadian period was significantly shortened (Fig. 7b; Table 3) to virtually match that of the bear’s own in vivo activity and temperature rhythms during winter dormancy (p > 0.05 for both; Tables 1 and 2). Bear serum also resulted in a significant increase in the amplitude of the Bmal1 rhythm by approximately 3-fold, irrespective of season (Fig. 7a,c; Table 3).

**Table 3:** Circadian characteristics of *Bmal1:luc* rhythms in bear fibroblasts obtained from winter dormant bears and exposed to fetal bovine serum (FBS) or bear serum from different seasons

Bear ID	Period (h)			Amplitude (cnts/s)		
	FBS	Dormant Serum	Active Serum	FBS	Dormant Serum	Active Serum
Juv#1 [23.8]^†^	24.7 ± 0.14	23.75 ± 0.07	23.3 ± 0	240 ± 4.21	712 ± 36	571 ± 164
Juv#2 [24]	23.9 ± 0.28	23.05 ± 0.07	23.7 ± 0	273 ± 54.9	1124 ± 58	1008 ± 223
Juv#3 [23.7]	24.5 ± 0.28	23.3 ± 0	23.15 ± 0.07	168 ± 17.9	471 ± 28	514 ± 63
Juv#4 [23.7]	24.5 ± 0.21	23.45 ± 0.07	23.05 ± 0.21	167 ± 18.07	445 ± 29	528 ± 83
Average	24.39 ± 0.13^a^	23.44 ± 0.12^b^	22.94 ± 0.11^c^	234 ± 24^a^	781 111^b^	749 ± 101^b^

^†^Values in brackets represent the period (h) of the same bear’s winter dormant activity rhythm in DD for comparison

^a,b^Different letters within a row are significantly different from each other, *p* ≤ 0.05

Values are means ± SEM

## Discussion

Using a variety of experimental approaches in captive bears and observations of naturally denning bears, we now provide the most compelling evidence to support the operation of a circadian clock during winter dormancy in brown and black bears. This was confirmed first by demonstrating that activity and Tb rhythms free-ran in constant conditions, a feature that also reveals their endogenous nature. Second, the biological clock was reset or shifted when exposed to single pulses of light applied at discrete times of the circadian cycle. Third, daily symmetrical light exposure caused activity bouts to coalesce and assume a 24 h periodicity. Fourth, fibroblasts obtained from dormant bears expressed robust clock-gene rhythms. Moreover, the period of this molecular rhythm closely matched that of the bear’s own activity and Tb rhythms but only under conditions where bear serum was used. Importantly, our findings of activity rhythms in captive bears were corroborated in wild bears. The present results are therefore entirely consistent with our earlier report of circadian rhythmicity in captive dormant bears [32] and together reveal that rhythmicity is a normal facet of torpid bear physiology.

Compared to the daily rhythms expressed in captive bears during the active (feeding) season, the activity rhythms of torpid bears were much weaker. They were however virtually identical in strength to their denning counterparts in the wild, indicating that the general conditions we used in our isolation experiments were comparable to those experienced by wild bears. Yet, based on the consistency of our findings it is surprising that others have not observed similar rhythms in hibernating bears, especially since those studies used bears exposed to environmental light and temperature fluctuations [20, 21] as some of our captive (ambient) and wild bears were. Aside from a species difference (black vs. grizzly), which seems unlikely given that we saw rhythms in a hibernating wild black bear, at least three possible explanations for this difference can be envisioned, although they are not necessarily mutually exclusive. The first relates to the low amplitude/strength of the activity rhythms in our captive bears (5–10 %), which if present in earlier studies may have been associated with correspondingly low temperature rhythms and thereby escaped detection due to methodological differences. Arguing against this is the fact that our temperature rhythms were actually more robust than the activity rhythms and were easily detected even when we used identical analytical methods as the earlier studies (e.g., Lomb-Scargle periodogram; data not shown) [21, 32]. Second, subcutaneous Tb rhythms could reflect a fundamentally different output of the circadian clock than core Tb measured using intraperitoneal implants. We also view this as unlikely since activity, a faithful reflection of master clock integrity, remained rhythmic and was phase-locked to temperature, even under different photic conditions. The third possibility relates to differences in den temperatures between studies. In the case of the Alaska and Wyoming black bears, outside temperatures and corresponding den temperatures frequently dropped below 0 °C for extended periods [20, 21] causing bears to shiver [21]. Thus, shivering may have masked an underlying circadian Tb rhythm as a result of the bear’s need to thermoregulate. Since our bears were held at a constant 7 °C and had relatively large body masses (>125 kg) when entering winter dormancy, this presumably placed them above a Tb that eliminated the need for shivering [21], allowing rhythms to be unmasked. This hypothesis however, remains to be tested. A final possibility, related to the third, is that poor body condition upon entering the den could have masked or suppressed Tb rhythms in an effort to conserve energy. This is based on observations from a single juvenile wild female brown bear (213052006) who had only an estimated 8.4 % body fat in August at the time of capture. This bear entered the den in November (adiposity unknown) and promptly ceased to exhibit any clear rhythmicity (Additional file 2: Figure S4). Then, in March, rhythmicity reappeared coincident with dramatic re-entrainment to the daily light: dark cycle visible as daily delays in activity onset until activity bouts were fully synchronized to dawn and dusk around the end of April. Although not conclusive, the data from this single case are indicative of a somewhat earlier than normal den exit perhaps due to the depletion of fat reserves and the need to obtain food.

We were also able to confirm in bears another feature of the circadian clock found in many other species [42–44], namely, a longer circadian period in older animals compared to younger ones. Previous studies had shown that dramatic changes in circadian period occurred around the time of puberty whereupon the clock “slows” leading to an adult circadian period that is generally longer than that of juveniles [44], consistent with our results. Because our younger bears had just entered their fourth year and therefore had not reached full sexual maturity [45] our comparisons are between different aged bears; thus, we cannot ascribe the change specifically to sexual maturation or to a particular hormone. However, future studies could address this by measuring reproductive hormones in blood in combination with estimating circadian period under constant conditions in a captive setting. The differences in rhythm period among life stages of bears could have interesting consequences during winter dormancy. For example, female grizzly bears den with cubs from birth and for several winters afterwards [45–48]. It is conceivable therefore that a female and cubs in a family group denning together in constant conditions would drift out of phase with one another by about 10–15 min per day (based on our current circadian estimates) and in opposite directions. Over time, the net effect of this drift would be overall activity in the den appearing virtually continuous and possibly arrhythmic, eventually returning to a rhythmic condition, and so on. Whether the cubs then influence the mother’s activity rhythm or the cubs are masking the mother’s rhythm is unclear; however, examination of the pre- and post-parturition activity patterns of the two pregnant wild bears in our study (Additional file 4: Figure S2) clearly reveals a reduction in amplitude and virtual arrhythmicity of the mother bear (Fig. 3b,c). This apparent loss of rhythmicity due to reductions in activity corroborates previous findings made in pregnant bears [49]. Additional analysis of hibernating family groups consisting of older cubs would be required to determine whether this phenomenon is limited to cubs-of-the year or is a general feature of denning female bears with offspring.

Responsiveness to an environmental synchronizing cue, such as light, is a hallmark of circadian systems. This was confirmed during winter dormancy in the current study by robust light-induced phase shifts in captive bears as well as light-associated activity changes in denning wild bears. Together, these observations expand our understanding of the behavioral ecology of bears to now include a dynamic responsiveness to a relevant environmental cue, even while dormant. The general pattern of responses to light, i.e., activity onsets delayed when exposed during the inactive period and advanced in the active period, generally mimic those seen in other mammals [50]. However, the marked effect of light to reset the clock to early morning irrespective of when it was applied during the inactive period was unexpected [50] for two reasons. First, the light intensity was low (~200lux). Second, the light duration that caused the largest phase shifts was often the shortest (1 h vs. 4 h). Collectively, these results suggest that bears are exquisitely sensitive to light during hibernation, itself rather surprising since during the active season bears use food to more effectively organize their behavior than light [32]. This apparent shift however could explain their temporal flexibility [51]. Indeed, only short daily (1 h) pairs of light pulses were sufficient to accomplish entrainment, which is consistent with extensive work done in other species [52]. Our findings in wild bears also appear to support these conclusions. However, a remaining question pertinent to denning wild bears is whether they shift their body position towards/away from a light source as a behavioral adaptation or if light exposure occurred incidentally. Given that our wild bears had light sensors affixed to collars on the neck, it is possible that the eyes were facing away from the light source when exposure occurred.

The maintenance of circadian rhythmicity and light entrainment by bears during winter dormancy may be important to optimize metabolic function. For example, metabolic rate could be directly proportional to circadian strength and could explain why rhythms disappeared (or were masked) in bears from earlier studies, i.e., their metabolic threshold was reached. In fact, the reduction in rhythm strength we observed in torpid bears(~50 %) is similar, but not as low as the metabolic suppression (60–75 %) previously reported for bears [5, 53, 54] suggesting that the putative threshold may not have been reached in our bears. Alternatively, even a weak (low amplitude) circadian rhythm may be necessary to maintain lower metabolic rate until challenged with extreme thermal or other demands. This would not be entirely surprising given the mounting evidence indicating that loss of rhythmicity and desyncrhony are associated with adverse metabolic outcomes [34, 37, 55]. Indeed, recent evidence suggests that rhythmicity in biological gene expression networks of eukaryotes as diverse as yeast, fruit flies, and mice serve to optimize metabolic costs, in part by modulating rhythm amplitude [38]. Thus, it is likely that a threshold of circadian amplitude exists beyond which the presumed cost/benefit relationship is lost. For example, it’s possible that a torpid, anorectic, denning female bear loses rhythmicity when faced with the additional metabolic demands of lactation. Similarly, a bear entering the den in poor body condition (low adiposity and unable to produce cubs if female [56]) might dispense with circadian rhythmicity altogether to maximize survival. Irrespective of these possibilities, the reduction in rhythm amplitude we observed, rather than a complete loss of rhythmicity could be viewed as evidence to support a metabolic optimization strategy. Nevertheless, precisely how rhythmicity, Tb and metabolic costs in shallow heterotherms are related remains to be determined.

Using data obtained from a small number of wild denning bears fitted with light sensors we were able to determine that those bear dens received periodic light exposure despite significant snow cover (based on environmental monitoring station, camera data at den sites and site visits to the U.S. black bear den). We now can confirm that wild hibernating bears are exposed to the same stimulus – light (albeit of different intensities), that was used in our captive studies to shape activity and Tb patterns. Thus, winter dormancy in bears provides a valid, physiologically relevant, condition in which to further explore the influence of light on the circadian clock independently of the confounding influence of food entrainment [32] and over many months. This ability to explore clock function without the contaminating influence of food anticipatory activity [57] holds great promise for our basic understanding of how central and peripheral clocks are organized.

Overall, our behavioral and physiologic results provide strong support for the existence of a functional circadian clock in torpid bears during winter dormancy. Although the Tb rhythms differ significantly from daily torpor bouts seen in some birds [58], they do suggest a lower Tb set point in torpid bears and one that is defended. Indeed, earlier findings in black bears by Tøien [5] were interpreted to indicate that Tb cycles were the result of a “regulated” process, distinct from a passive hypothermic response [59]. The similar maximum daily Tb of our bears housed in constant temperature versus the bear exposed to natural temperature changes would support this conclusion. Thus, these earlier findings combined with our current ones would suggest that bears are capable of maintaining a tightly regulated torpor around a lower Tb set point and over multiple timescales. Together, these features appear to place winter dormant bears somewhere between “true” hibernators and shallow heterotherms [59].

A recognized caveat to the interpretation of activity and Tb rhythms is that these measures represent ‘outputs’ of a central (brain) clock and are therefore subject to misinterpretation as a result of masking or other factors. Thus, it is necessary to confirm clock operation in other ways, such as by examining the molecular clock directly. Circadian clocks are distributed in tissues throughout the body [60] and these provide a readily available means to assess clock operation [61]. We therefore collected skin fibroblasts from winter dormant bears and infected these cells with a lentiviral clock gene (*Bmal1*) construct linked to luciferase enabling a real-time luminescent readout in vitro [61]. Our findings of robust rhythms in fibroblasts obtained from the same animals whose activity and Tb rhythms were confirmed during winter dormancy now provides direct proof that a functioning biological clock is an inherent feature of dormant grizzly bear physiology. Our findings stand in stark contrast to those in European hamsters (*Cricetus cricetus*) whose clock “stops” during hibernation [19] and to arctic reindeer (*Rangifer tarandus*), who don’t hibernate, but lose their behavioral and molecular rhythms in winter when measured using similar techniques to ours [62]. Somewhat to our surprise, the period of bear fibroblast rhythms required the bear’s own serum to match that observed in vivo. These results suggest that humoral factors may play additional roles in maintaining circadian integrity of the entire metabolic engine of these animals. Because we performed our culture experiments at 37 °C and since lower culture temperatures also appear to influence fibroblast circadian period in a homeotherm [63] it remains to be determined what influence different culture temperatures and serum combinations have on these bear rhythms.

## Conclusion

In conclusion, we provide both in vivo and in vitro evidence supporting the expression of circadian rhythms in bears during winter dormancy. These findings, along with earlier work in grizzly bears [64] and polar bears add to the mounting evidence that these closely related species may exhibit an evolutionarily advanced form of torpid biology [65, 66].

## Methods

### Animals

Captive grizzly bears were housed at the Washington State University (WSU) Bear Research, Education and Conservation center as described previously [32]. Bears of both sexes were used and ranged in age from 2 to 12 years and weighed between 127 and 331.3 kg (Additional file 1: Table S1) at the time of winter dormancy. Bears younger than 5 years old were considered juveniles. Environmental light and activity data were also obtained from two wild grizzly bears in Alberta, Canada (2013), one black bear in Idaho in 2011 and seven wild grizzly bears (2011–2013) in Montana, USA.

Captive bears entered winter dormancy following the naturally declining appetite and withdrawal of all food in late October. This is typically followed by a >90 % decrease in activity (standing) in early-mid December [41]. In six of the same captive bears used in the current study Tb was also measured during both the active and dormant seasons (see Results) using a digital rectal thermometer. Once dormant, the captive bears were moved to an isolation building where constant conditions of temperature and light could be maintained [32]. Bears in isolation were housed individually in culvert type enclosures [32] from November to March in each of three consecutive winter dormancy seasons (2012–2013, 2013–2014, and 2014–2015). The internal dimensions of the enclosures (LxWxH) measured 2.4 x 1.2 x 1.2 M. Enclosures were provided with elevated grating and drainage and all bears were provided water *ad libitium*. No food was provided. All isolation studies began with constant darkness (DD) followed by one or more light applications (detailed below). When not housed in isolation, bears were housed in larger enclosures and exposed to ambient light and temperature fluctuations (for details see [32]). Wild bears were captured, fitted with collars containing GPS transmitters and accelerometers according to accepted standards. Wild grizzly bears in Alberta were fitted with Followit® GPS iridium satellite collars. These collars were programmed to record locations every hour during the non-denning period and once in the den locations were attempted daily until den emergence. In Montana and Idaho, wild bears were fitted with Telonics GPS collars (Models 4500 and 4590 Telonics, Mesa, AZ). The collars were programmed to record locations every two hours during the non-denning period. Wild bears were allowed to enter winter dormancy naturally and were left undisturbed by the researchers. Body weights of wild bears at the time of den entry were not determined.

### Activity and Tb monitoring

Captive bears were fitted with Actical® dual axis accelerometers (Phillips Respironics, Bend, OR) capable of capturing body movement with high fidelity and over long periods [32]. These lightweight devices were housed in a protective aluminum case that was glued to the bear’s fur in the lateral aspect of the neck/shoulder region using two-part epoxy. We collected movement data at 1 min intervals beginning in early fall until the end of winter dormancy (2013–2015) or only during winter dormancy (2012). For three wild bears (two brown, one black) captured in Canada and U.S. we used a different accelerometer (Actiwatch Spectrum®, Phillips) capable of measuring both activity and white light (sampling rate - 32Hz). These were housed in a custom designed weatherproof anodized aluminum case with clear acrylic window to facilitate light detection and were attached to the collar belting approximately 8 cm below the center line at the top of the GPS collar (Canada) or on the center line (U.S.). Montana and Idaho wild bears were also fitted with Telonics GPS collars containing three-axis accelerometers that recorded activity year-round. We collected Actiwatch activity and light data at 5 min intervals to extend battery life and enabling data to be collected for most of the winter dormancy period. The activity data from accelerometers housed within the U.S. GPS collars were also collected at 5 min intervals and in one case were collected over multiple years. Accelerometer data for all devices are interpolated in real-time to yield a single integrated value (counts) per sampling interval.

Tb data were collected in captive bears using data loggers (iButton, DS1922L; Maxim Integrated, Inc., San Jose, CA) implanted subcutaneously in the axial region. Prior to implanting the iButtons were warmed to 50 °C, dipped in M-coat W1 wax (Vishay Precision Group, Malvern, PA) and allowed to dry. The dipping process was repeated 5 times followed by cold (gas) sterilization. Bears were anesthetized and the implant site prepared as described below for biopsy sampling. Bears tolerated the coated loggers well as indicated by minimal scar tissue forming at the implant site, absence of adhesions, and no evidence of irritation. Once the studies were completed, bears were re-anesthetized, the loggers were removed, and the skin re-sutured. Tb was also measured during the active season and winter dormancy in six anesthetized captive bears that had not been implanted with loggers at the time. For this, a digital rectal thermometer was used while the bears were undergoing unrelated procedures. Three Tb measurements were taken over approximately a one-hour time period and the average temperature determined. Temperature logger data were collected from each bear at 30 min intervals. One bear housed under ambient conditions also received a temperature logger. Identically prepared iButtons were also placed in the isolation building housing the bears to record Ta and at the same frequency. Temperature loggers and digital rectal thermometer were calibrated against a mercury thermometer using a water bath. Sensitivity of the temperature loggers was programmed for 0.0625 °C. The resultant accuracy of both devices was determined to be ±0.1 °C.

### Lighting and photocycles

Full spectrum fluorescent bulbs (GE; 32 W, 6500 K, model F32T8 SP65 ECO) 2 bulbs/isolation chamber) were used to provide approximately 200 lux of light at the bear’s eye level, roughly 2 meters from the light source. Light intensity naturally varied depending on the bear’s position with the chamber. To generate phase-response curves, light was applied in pulses ranging from 1–4 h in duration depending on the year of the study. In the first year, a light regimen consisting of 1 h light (L) interspersed with 11 h of darkness (D) was applied to explore entrainment (i.e. synchronization to a daily photocycle (1 L:11D:1 L:11D). This photocycle was maintained for 40 days. In year two, a single light pulse of 4 h duration was applied. This was followed by two 1 h pulses each 30 days apart and at fixed times of the day for all bears although the exact circadian time varied depending on when the light was applied and the bear’s own free-running period (see below). In year three, only a single 4 h pulse was used and this was applied at the same time of day for all bears. These photocycles are similar to those used in many previous studies in other species to probe circadian and entrainment properties [52].

### Data analysis

Activity and temperature data were analyzed using custom MATLAB scripts written to run the computations, and making use of two freely available toolboxes: JLAB [67] and WMTSA [68]. JLAB was employed to compute the analytic wavelet transform (AWT) scalogram and ridges using the Morse wavelet function with β = 10 and γ = 3; see Lilly & Olhede [69] for further details. WMTSA, companion software to the book [70], was used to compute the translation-invariant discrete wavelet transform (DWT) with the Daubechies least-asymmetric filter of length 12. All calculations were run in MATLAB R2015a (The MathWorks, Natick, MA). MATLAB scripts to run the computations are available on request. See [71, 72] for details on wavelet analyses.

Because we applied light pulses at fixed times with no knowledge of the bear's circadian phase at which they were applied until the experiment ended, we needed a method to accurately determine circadian time (CT). To this end, CT6 was defined as activity peak (CT0 = onset of activity, assuming ~ 12 h period of activity) and then mapping this phase onto at least 10 consecutive days of activity prior to applying a light pulse. The number of days (range = 10–20) varied depending on the stability of the rhythm expressed in DD. Light-induced phase shifts were then identified by mapping the same phase (CT6) onto 2–4 days of data following light application at which point the time-difference between the pre- and post-light phases was calculated. By convention, negative values reflect phase delays whereas positive values reflect phase advances [52]. Phase shifts were only determined for activity due to the discrete nature of the data.

All computations applied to activity and temperature records were scaled to 15-min bins. Period was calculated using three different methods for comparison: (1) maximum entropy spectral analysis (MESA) of the mean-subtracted time series as described in [73]; (2) fit of the DWT circadian component (12–64 h period band) to extract period and phase that were also used to compute the phase shifts in response to a light pulse; (3) the mean over the AWT period ridge for the portion of the ridge falling between periods 20 and 28 h with magnitude of at least half the median value and at least 1.5 days from the edges (to avoid edge effects). The debiased AWT ridge coefficients were also used to assess the rhythm’s amplitude. Circadian strength equals the proportion of variance in the 12–64 h period band and was determined from the DWT circadian component of the time series. Acrophase was computed from the peaks of the DWT circadian component of the time series. The tempogram display of the temperature record shows the positive portion of the summed circadian details covering the 0.5–32 h frequency band.

The comparative analysis of activity and Tb also involved multiple methods. The phase difference between the rhythms was assessed in three ways: (1) using the sine-fit period and phase of the DWT circadian components (12–64 h period band), (2) the lag corresponding to the first peak of the cross-correlation of the DWT circadian components (4–64 h period band), and (3) the phase of the peak spectral coherence of the mean-subtracted time series. The spectral coherence is defined as the magnitude-squared coherence function (square of the Fourier transform of the cross-correlation divided by the product of the Fourier transforms of the autocorrelations of the two time series) using Welch's overlapped averaged periodogram method and was computed using MATLAB’s *mscohere* and *cpsd* with a Hamming window of 2 weeks.

### Cell culture

Four juvenile bears were anesthetized during winter dormancy as previously described [32]. An area of the rump was shaved and surgically prepared. A small skin sample was collected using a 6 mm biopsy punch and immediately placed in Dulbecco’s Modified Eagle’s Medium (DMEM) supplemented with 4500 mg/l glucose, 50 % Fetal Bovine Serum (FBS) and 1 % pennicillin/streptomycin solution at 4 °C until processed as follows. Each tissue sample was disrupted mechanically by trituration and the dispersed cells incubated in warm DMEM supplemented with 20 % FCS, 1/100 amphotericin B (Sigma A2942) and 0.2 ml Liberase Blendzyme 3 at 37 °C and 5 % CO_2_ for about 6 h. The digested tissue was then washed in phosphate buffered saline (PBS) and re-suspended in DMEM with 20 % FCS and amphotericin B, and placed under a Millipore Millicell CM membrane disc and left overnight at 37 °C. On the following day, the membrane was removed, the cells were washed with PBS, and incubated in DMEM supplemented with either 10 % FCS, 10 % bear dormant serum or 10 % bear active season serum, all containing 1 % Penicillin/Streptomycin, and L-glutamine and cultured overnight. The cells were then infected with a mouse *Bmal1-luciferase* encoding lentivirus, synchronized with dexamethasone and the luminescence measured for 5 days at 37 °C using a luminometer as described previously [61].

### Statistical analysis

Group-wise comparisons were made by one-way Analysis of Variance or Kruskal-Wallis test where appropriate. Post-hoc comparisons were made following Holm-Sidak correction. Statistical analyses were performed using Prism 6.0 (Graphpad Software, Inc., La Jolla, CA). P values ≤ 0.05 were considered statistically significant.

## Additional files

Additional file 1:
**Table S1.** Body weights (kg) of captive bears just prior to entering winter dormancy. (PDF 630 kb) Additional file 2:
**Figure S4.** Actograms (A) and scalograms (B,C) of a wild female grizzly bear during early denning (winter 2012-B) and mid-winter dormancy to den exit (2013-C) illustrating the lack of rhythmicity and subsequent re-appearance of locomotor rhythmicity prior to and at the time of den emergence (arrow). Day 71 corresponds to Nov. 8, 2012 in panel B. Day 1 corresponds to Jan. 1, 2013 in panel C. * - first day of noticeable entrainment (2012); Arrow – date of den exit based on GPS location fix. (PDF 630 kb) Additional file 3:
**Figure S1.** A. Relationship between activity counts and light intensity for three denning wild bears. B. Relationship between time of light exposure and time of the largest activity bout in the same three bears shown in A. All times local. (PDF 109 kb) Additional file 4:
**Figure S2.** Frequency distribution of light exposures (minutes at >5lux) for three wild bears fitted with activity and light sensors. Data are grouped into 10 min bins. (PDF 631 kb) Additional file 5:
**Figure S3.** Daily (24 h) activity profiles and scalograms of activity data from two denning pregnant wild bears (A – black bear; B – grizzly bear) before and after birth. Note the general reduction (and eventually loss) of rhythms after parturition. Activity in Panel B post-birth is artificially elevated due a voltage offset being introduced as the battery power was declining in this Actiwatch. (PDF 627 kb)
