# Supplementary figures and images for: The bear circadian clock doesn’t ‘sleep’ during winter dormancy
Source: Front Zool. 2016 Sep 17;13:42. doi: 10.1186/s12983-016-0173-x (PMC5026772; doi:10.1186/s12983-016-0173-x)

FIGURE S1

Supplemental Information

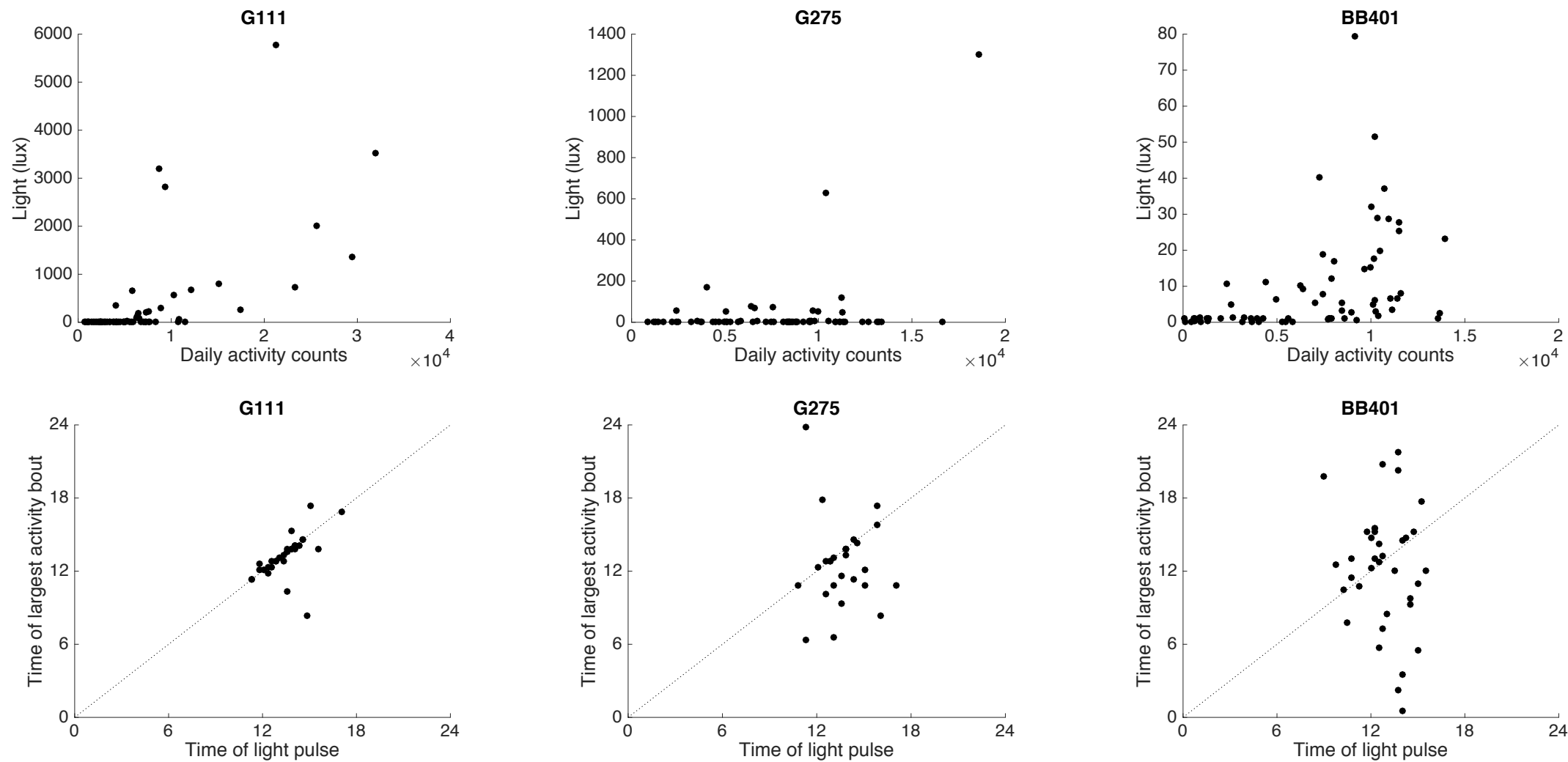

Supplement: Additional file 1: — Table S1. Body weights (kg) of captive bears just prior to entering winter dormancy. (PDF 630 kb) [file 12983_2016_173_MOESM1_ESM.pdf]

FIGURE S2

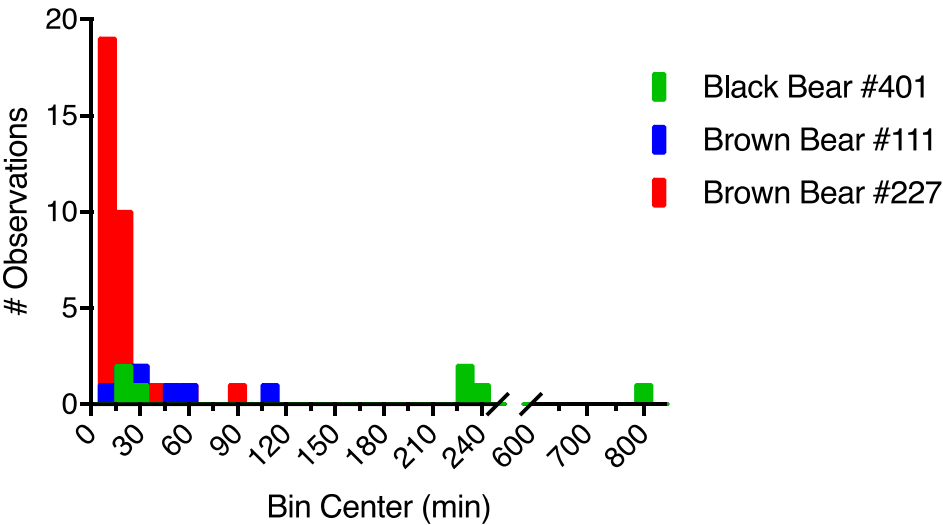

Supplement: Additional file 2: — Figure S4. Actograms (A) and scalograms (B,C) of a wild female grizzly bear during early denning (winter 2012-B) and mid-winter dormancy to den exit (2013-C) illustrating the lack of rhythmicity and subsequent re-appearance of locomotor rhythmicity prior to and at the time of den emergence (arrow). Day 71 corresponds to Nov. 8, 2012 in panel B. Day 1 corresponds to Jan. 1, 2013 in panel C. * - first day of noticeable entrainment (2012); Arrow – date of den exit based on GPS location fix. (PDF 630 kb) [file 12983_2016_173_MOESM2_ESM.pdf]

FIGURE S4

**A**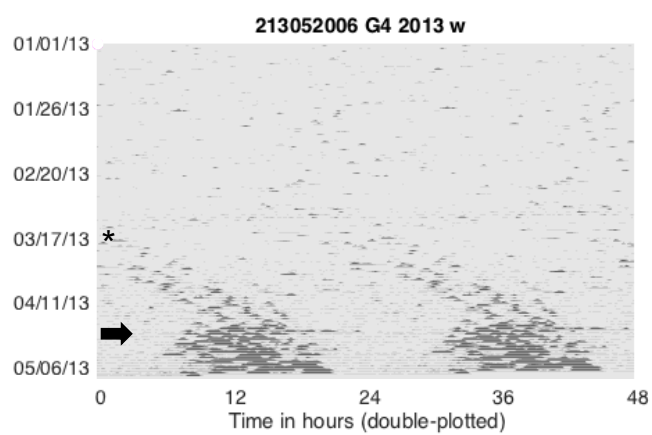**B**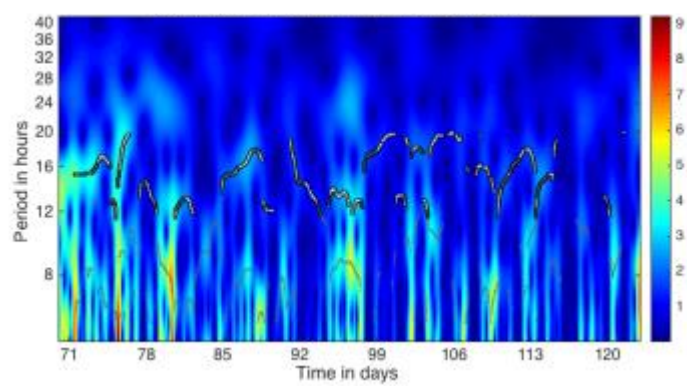**C**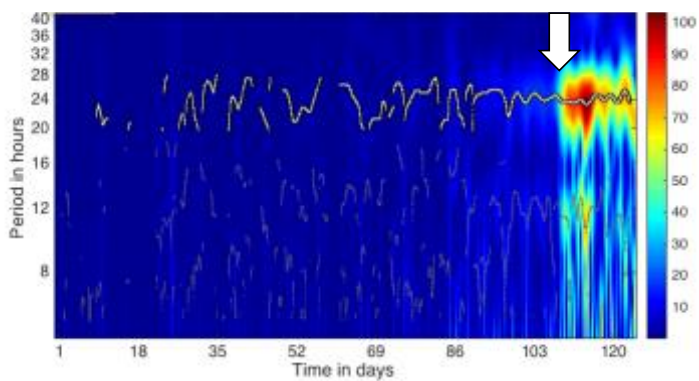

Supplement: Additional file 4: — Figure S2. Frequency distribution of light exposures (minutes at >5lux) for three wild bears fitted with activity and light sensors. Data are grouped into 10 min bins. (PDF 631 kb) [file 12983_2016_173_MOESM4_ESM.pdf]
