# Supplementary material for: The bear circadian clock doesn’t ‘sleep’ during winter dormancy
Source: Front Zool. 2016 Sep 17;13:42. doi: 10.1186/s12983-016-0173-x (PMC5026772; doi:10.1186/s12983-016-0173-x)
Supplement: Additional file 5: — Figure S3. Daily (24 h) activity profiles and scalograms of activity data from two denning pregnant wild bears (A – black bear; B – grizzly bear) before and after birth. Note the general reduction (and eventually loss) of rhythms after parturition. Activity in Panel B post-birth is artificially elevated due a voltage offset being introduced as the battery power was declining in this Actiwatch. (PDF 627 kb) [file 12983_2016_173_MOESM5_ESM.pdf]

Table S1. Body weights (kg) of captive bears just prior to entering winter dormancy.

Bear ID (sex) and Age Class	***Period of Dormancy***		
	**2012-2013***	**2013-2014***	**2014-2015***
J1 (m)	127.3	179.4	185.5
J2 (m)	127	151.3	171
J3 (m)	163.3	177.4	-
J4 (m)	137.4	156	176
J5 (m)	ND	-	-
**Average ± SD**	138.8 ± 17.1	166 ± 14.4	177.5 ±7.4
A1 (m)	331.3	324.8†	-
A2 (m)	-	242.2	265.4
A3 (f)	217	150.1	-
A4 (f)	189.1†	126.3	-
A5 (f)	187.3†	-	-
A6 (f)	254.4†	-	-
A7 (f)	245.8†	-	-
A8 (f)	259.7†	-	-
**Average ± SD**	240.7 ± 49.8	210.9 ± 90.9	265.4

* Weighing dates: 2012 – Oct. 27; 2013 – Nov. 1; 2014 - Nov. 2
†These captive bears were housed under ambient conditions
J, A - juvenile, adult
ND – not determined
